# Clinical Significance of Electronegative Low-Density Lipoprotein Cholesterol in Atherothrombosis

**DOI:** 10.3390/biomedicines8080254

**Published:** 2020-07-30

**Authors:** Chih-Sheng Chu, Shi Hui Law, David Lenzen, Yong-Hong Tan, Shih-Feng Weng, Etsuro Ito, Jung-Chou Wu, Chu-Huang Chen, Hua-Chen Chan, Liang-Yin Ke

**Affiliations:** 1 Center for Lipid Biosciences, Kaohsiung Medical University Hospital, Kaohsiung Medical University, Kaohsiung 807377, Taiwan; chucs@kmu.edu.tw; 2 Division of Cardiology, Department of International Medicine, Kaohsiung Medical University Hospital, Kaohsiung 807377, Taiwan; 3 Division of Cardiology, Department of Internal Medicine, Kaohsiung Municipal Ta-Tung Hospital, Kaohsiung 80145, Taiwan; 4 Department of Medical Laboratory Science and Biotechnology, College of Health Sciences, Kaohsiung Medical University, Kaohsiung 807378, Taiwan; shlaw_0909@hotmail.com (S.H.L.); dav.lenzen@gmail.com (D.L.); yonghongtan96@hotmail.com (Y.-H.T.); eito@waseda.jp (E.I.); 5 Department of Healthcare Administration and Medical Informatics, College of Health Sciences, Kaohsiung Medical University, Kaohsiung 807378, Taiwan; sfweng@kmu.edu.tw; 6 Department of Biology, Waseda University, Tokyo 162-8480, Japan; 7 Waseda Research Institute for Science and Engineering, Waseda University, Tokyo 162-8480, Japan; 8 Division of Cardiology, Department of Internal Medicine, Pingtung Christian Hospital, Pingtung 90059, Taiwan; wujcdd@gmail.com; 9 Vascular and Medicinal Research, Texas Heart Institute, Houston, TX 77030, USA; cchen@texasheart.org; 10 Graduate Institute of Medicine, College of Medicine, & Drug Development and Value Creation Research Center, Kaohsiung Medical University, Kaohsiung 807378, Taiwan

**Keywords:** electronegative low-density lipoprotein, LDL(–), L5 LDL, oxidized LDL, oxLDL, cardiovascular disease, atherosclerosis

## Abstract

Despite the numerous risk factors for atherosclerotic cardiovascular diseases (ASCVD), cumulative evidence shows that electronegative low-density lipoprotein (L5 LDL) cholesterol is a promising biomarker. Its toxicity may contribute to atherothrombotic events. Notably, plasma L5 LDL levels positively correlate with the increasing severity of cardiovascular diseases. In contrast, traditional markers such as LDL-cholesterol and triglyceride are the therapeutic goals in secondary prevention for ASCVD, but that is controversial in primary prevention for patients with low risk. In this review, we point out the clinical significance and pathophysiological mechanisms of L5 LDL, and the clinical applications of L5 LDL levels in ASCVD can be confidently addressed. Based on the previously defined cut-off value by receiver operating characteristic curve, the acceptable physiological range of L5 concentration is proposed to be below 1.7 mg/dL. When L5 LDL level surpass this threshold, clinically relevant ASCVD might be present, and further exams such as carotid intima-media thickness, pulse wave velocity, exercise stress test, or multidetector computed tomography are required. Notably, the ultimate goal of L5 LDL concentration is lower than 1.7 mg/dL. Instead, with L5 LDL greater than 1.7 mg/dL, lipid-lowering treatment may be required, including statin, ezetimibe or PCSK9 inhibitor, regardless of the low-density lipoprotein cholesterol (LDL-C) level. Since L5 LDL could be a promising biomarker, we propose that a high throughput, clinically feasible methodology is urgently required not only for conducting a prospective, large population study but for developing therapeutics strategies to decrease L5 LDL in the blood.

## 1. Introduction

Blood cholesterol remains the critical therapeutic target for primary and secondary prevention in clinical atherosclerotic cardiovascular disease (ASCVD), according to the international guidelines published by the American College of Cardiology (ACC) and the American Heart Association (AHA) [1,2]. Incorporating both low-density lipoprotein-cholesterol (LDL-C) and high-density lipoprotein-cholesterol (HDL-C) as the essential parameters, many proposed risks calculators, such as Pooled Cohort Equation or ASCVD Risk Estimator Plus, are currently available for assessing a person’s overall risk and monitoring statin therapy [1]. Some reports advocate “the lower the LDL-C, the better” [3], yet other studies criticized that this cannot preclude the occurrence of ASCVD but instead brings more individuals to suffering the side effects after statin exposure, such as muscle pain, impaired liver function, and new-onset diabetes mellitus [4,5], adversely impacting the quality of life.

We previously reported that by using fast-protein liquid chromatography, LDL can be divided into five subfractions, L1-L5, based on increasing electronegativity. Of those, L5 LDL exhibits atherothrombogenic and proinflammatory properties in vitro and in vivo [6]. The concentration of L5 LDL is low in normal healthy subjects [7], but increased in patients with chronic cardiometabolic disorders (e.g., type 2 diabetes [8], metabolic syndrome [9]) or acute ischemic events (e.g., ST-elevation myocardial infarction [10], ischemic stroke [11]), regardless of their plasma LDL-C concentrations (Table 1). More recently, we demonstrated that L5 LDL also plays an atherogenic role in patients with systemic lupus erythematosus (SLE) as well as rheumatoid arthritis (RA), who often have severe atherosclerotic complications that, however, cannot be attributed to conventional risk factors [12,13].

In 2018, Chu et al. determined the cut-off values of L5 levels for clinical ASCVD using receiver operating characteristic (ROC) curve analysis [7]. In their original study, individuals with a plasma L5 LDL level of less than 1.7 mg/dL showed no clinical evidence of ASCVD. In contrast, individuals with the plasma L5 LDL concentration exceeding the range of 2.3~2.6 mg/dL exhibited subclinical atherosclerosis or coronary artery disease (CAD) even when LDL-C or triglyceride (TG) levels were not elevated [7,13]. While statin can reduce L5 LDL in quantity by lowering the total LDL volume, their mechanism of action is not in quality targeting on the clearance of L5 LDL [14]. Discontinued statin therapy results in rebounds of both total LDL-C and L5 LDL to the pretreatment levels in three months [15]. These findings indicate that L5 LDL should be a potential clinical biomarker to be accurately measured for ASCVD risk stratification. We hereby propose a plasma L5 LDL concentration of greater than 1.7 mg/dL as a therapeutic threshold of initiating lipid-lowering treatments, on the basis that the odds ratio for CAD would reach 17.68 [7]. Furthermore, precision medicine targeting the removal of L5 LDL is of great importance in the future.

In this review, we summarize the latest research advances in the field of L5 LDL cytotoxicity and pathogenic significance, with the goals of (1) identifying L5 LDL as the primary biomarker for clinical ASCVD risk and the guide for statin or other lipid-lowering therapies; (2) establishing the recommended therapeutic threshold of plasma L5 LDL; (3) encouraging further research in the development of rapid quantification of L5 LDL for large scale epidemiological surveys among different cohorts; (4) providing insightful information for research on the new therapeutic strategies targeting L5 LDL or its atherogenic moieties. The most recent advances, notably the newly-established connection to autoimmune vascular diseases, actively support L5 LDL as a promising new domain of lipoprotein research.

## 2. Characteristics of Electronegative Low-Density Lipoprotein (L5 LDL) 

### 2.1. Definition and Methodolgy

**Figure 1:**
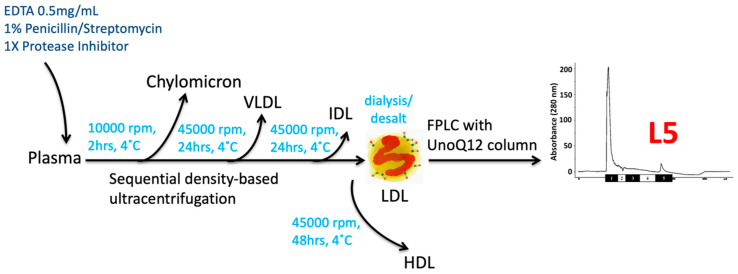
Schematic procedures of L5 LDL isolation. EDTA, antibiotics, and protease inhibitors are materials for the prevention of protein degradation. Samples undergo sequential density-based ultracentrifugation (10,000 rpm at 4 °C for 2 h; d = 1.004, 45,000 rpm at 4 °C for 24 h; d = 1.019, 45,000 rpm at 4 °C for 24 h; d = 1.063, 45,000 rpm at 4 °C for 48 h), and after that, LDL (d = 1.019~1.063) can be purified. Additional three times dialyzed against TRIS/EDTA buffer at pH 8.0 and later sterilized by 0.22 µm filter, the LDL sample can be further isolated into five subfractions by a fast-protein liquid chromatography (FPLC) system equipped with an UnoQ12 column. L5 LDL is the most electronegative subfraction. VLDL: very-low-density lipoprotein; IDL: intermediate-density lipoprotein; LDL: low-density lipoprotein; HDL: high-density lipoprotein; L5: electronegative LDL; FPLC: fast-protein liquid chromatography; rpm: revolutions per minute; 1× Protease Inhibitor: cOmplete™ (Roche Diagnostics, Basel, Switzerland).

The concept of “electronegative LDL” was first proposed by Gotto and Hoff at Baylor College of Medicine in 1979. They purified the lipoprotein from human aortic plaques and normal intima by using differential ultracentrifugation. Their immunoelectrophoresis results showed that a group of LDL-like particle from aortic extracts was more electronegative than plasma LDL and associated with the atherosclerotic progression [16]. In 1987, the presence of “modified LDL” was reported by Dr. Avogaro and his colleagues in Italy. They proposed that the atherogenic properties and endothelial cells (ECs) cytotoxicity of modified LDL can be enhanced due to the occurrence of lipoprotein oxidation [17]. Later in 1988, they used fast-protein liquid chromatography (FPLC) equipped with an ion-exchange column to separate plasma LDL into electropositive LDL(+) and electronegative LDL(–) subfractions [18]. LDL(–) particles are heterogeneous in morphology and size and have a tendency to aggregate by electronic microscopic exam. Electronegative LDL has emerged as a naturally occurring, atherogenic entity irrespective of the concentration of plasma LDL-C [19,20,21]. In 2003, Yang and Chen modified the protocol of separation, LDL can be chromatographically divided into five subfractions with increasing electronegativity, L1–L5 [19,20] (Figure 1). L5 LDL is the most electronegative subfraction.

### 2.2. Glycosylation of Apolipoproteins in L5 LDL

With the technique developed by Yang and Chen, the least electronegative subfraction of LDL is termed as L1, whereas the most electronegative LDL is L5 [20]. It can be more accurate because L1 does not appear electropositive. Besides, the intermediary subfractions (i.e., L2-L4) can be useful while investigating the transitional changes of electronegativity from L1 to L5. Based on the definition that one particle of LDL contains one mole of apolipoprotein B100 (apoB100), L5 LDL isolated from plasma contains many other proteins such as apo(a), apolipoprotein CIII (apoCIII), apolipoprotein J (apoJ), platelet-activating factor acetylhydrolase (PAF-AH), and paraoxonase 1 (PON1) [22,23], which are not in L1 LDL. Besides, L5 LDL has significantly higher levels of apolipoprotein E (apoE) and apolipoprotein AI (apoAI). 

In cardiomyocytes, apoE interacts with the voltage-dependent anion-selective channel (VDAC), leading to dynamin-related protein 1 (Drp1) phosphorylation and mitochondrial fission [24]. Additionally, apolipoproteins associated with L5 LDL from human plasma particles are found to be highly glycosylated. For instance, apoE that shows 94S, 194T, and 289T glycosylation with sialic acid terminal glycan, which alters the receptor selectivity and lipid-binding capability [25]. These findings may support that single nucleotide polymorphisms (SNPs) of apoE with changing electrical charges are associated with metabolic disorders [26,27]. ApoB100 glycosylation is also associated with the sphingomyelinase-like activity of electronegative LDL [28]. With sphingomyelinase activity, ceramide can be overproduced through the sphingomyelin hydrolysis pathway and therefore induces endothelial cell apoptosis.

### 2.3. Atherogenic Lipid Moieties of L5 LDL

By using colorimetric methods, L5 LDL shows triglyceride-rich but reduced cholesteryl ester in the lipid composition [19,29,30]. These findings match clinical observations regarding the higher plasma triglyceride content in patients with metabolic syndrome [31]. Other than that, L5 LDL from patients with familial hypercholesterolemia or diabetes has been shown to contain higher levels of lipoprotein-associated phospholipase A_2_ (Lp-PLA2) [32,33,34]. The function of Lp-PLA2 is to hydrolyze phospholipids and generate lysophosphatidylcholine (LPC) and non-esterified fatty acids (NEFA) [35]. 

By using mass spectrometry, L5 LDL shows higher levels of ceramide, lysophosphatidylcholine (LPC) and platelet-activating factor (PAF) in comparison to L1 LDL [12,28]. Ceramide plays an essential role in stress-related cellular responses and apoptosis [36,37,38,39]. Alterations in ceramide levels have been recognized in pathological conditions such as Alzheimer’s disease [40], type 2 diabetes [41], and cardiovascular diseases [42]. LPC stimulates inflammatory chemokine expression from endothelial cells [43,44,45,46], impairs arterial relaxation [47], increases oxidative stress [48,49], and inhibits endothelial cell migration and proliferation [50,51]. The level of LPC increases in cardiovascular diseases (CVDs), diabetes, and renal failure [52,53,54]. Besides, our recent studies also showed that LPC and PAF are inflammatory mediators that lead to the differentiation of monocytes into proinflammatory CD16^+^ cells and contribute to endothelial dysfunction and vascular aging, thereby providing a novel explanation for the early onset of atherosclerosis-associated complications [12,55]. 

## 3. Cellular Signaling of L5 LDL

### 3.1. Signaling in Endothelial Cells

Accumulating evidence suggests that L5 LDL interacts with multiple cells such as endothelial cells [8,15,56,57,58], platelets [10,11], monocytes [9,59,60,61,62,63], and cardiomyocytes [24,64,65,66,67]. L5 LDL attracts both monocytes and lymphocytes to endothelial cells (ECs) [58], indicating the contribution in the early stage of atherosclerosis. L5 LDL is not recognized by the LDL receptor (LDLR) [68], but rather, it signals through the lectin-like oxidized LDL receptor-1 (LOX-1) and platelet-activating factor receptor (PAFR) [10,20,57]. LOX-1, initially identified as the major receptor for oxLDL in ECs, is expressed at high levels in pro-atherogenic settings and has been shown to have a critical role in atherogenesis [69,70].

Upon internalization through LOX-1, L5 LDL induces TNF-α expression, which subsequently triggers the expression of new LOX-1, making surrounding vascular epithelial cells increasingly susceptible to damage and apoptosis [8]. In cardiomyocyte, L5 LDL can enhance the ECs’ activities by secreting Glu-Leu-Arg (ELR)^+^, lipopolysaccharide-induced CXC chemokine (LIX) and interleukin-8 (IL-8), which further initiated CXCR2/PI3K/NF-κB signaling. These signals will then contribute and induce cardiomyocyte apoptosis through the release of the proinflammatory cytokines TNF-α and IL-1β [64]. 

On the other hand, L5 LDL possesses the ability to impair vascular ECs integrity and induce ECs apoptosis by suppressing the fibroblast growth factor 2 (FGF2) transcription and disrupting its autoregulation repairing system [8]. Cellular exogenous FGF2 plays a pivotal role in promoting cell metabolism, proliferation, cell survival, growth, and preventing apoptosis through the PI3K-Akt pathway [71]. These findings indicate that ECs dysfunction can be augmented by disrupting the FGF2 formation.

### 3.2. Signaling in Platelets

In the field of platelets, adenosine diphosphate (ADP), one of the major soluble agonists can mainly regulate the P2Y12/phosphatidylinositol-3 kinase (PI3K) pathway for platelet aggregation [72]. Apart from that, ADP increases LOX-1 expression and glycoprotein (GP)IIb/IIIa activation [73]. Through LOX-1 and PAFR, L5 LDL enhances ADP signaling of platelets. Besides, L5 LDL increases P-selectin and tissue factor expression on ECs. Particularly, P-selectin shows to be capable of interaction with the PAFR and induces platelet adherence and activation. These platelet-EC interactions triggered by L5 LDL may promote thrombosis formation, leading to STEMI [10]. 

In another study, L5 LDL induces amyloid β (Aβ) secretion through LOX-1 and IkB kinase 2 (IKK2) activation. Synergistically, L5 LDL and Aβ promote the platelet aggregation and activation [11]. These findings suggest that L5 is the thrombogenic fraction of LDL and may contribute to platelet hyper-reactivity, STEMI, and stroke complications [74,75]. 

### 3.3. Signaling in Immune Cells

During the past three decades, the autoimmune hypothesis of atherosclerosis is prospering under the evidence of LDL-containing circulating immune complexes (LDL-CIC) accumulation in atherogenesis [76,77]. Different from native LDL, the LDL-CIC is more electronegative and may alter lipid and lipoprotein levels in ECs and macrophages [78]. Besides, LDL(–) can induce inflammatory cytokines to release from monocytes, such as monocyte chemoattractant protein 1 (MCP1), interleukin-6 (IL-6), IL-8, growth-related oncogene (GRO), granulocyte-monocyte-colony stimulating factor (GM-CSF) [79], matrix metalloproteinase-9 (MMP-9) and its inhibitor tissue inhibitors of metalloproteinase-1 (TIMP-1) [63]. The release of these cytokines by LDL(–) might be mediated through CD14/toll-like receptor 4 (TLR4) signaling pathways [80]. 

LDL(–) also involves apoptosis and cytokine induction by upregulation of proapoptotic factor Fas on mononuclear leukocytes [81]. Apart from that, Klimov et al. have reported that mouse macrophages cultured with the presence of LDL-CIC can increased uptake of LDL [82]. Additionally, incubation of human peritoneal macrophages with the same condition causes the transformation of macrophages into foam cells [83]. 

In our recent studies, L5 LDL triggers the differentiation of CD16^+^ monocytes. Through the CX3CR1 and CD16 expressing monocytes interact with CX3CL1-positive activated ECs, L5 LDL induces monocyte-endothelial cell adhesion [12]. L5 LDL also enhances the polarization of M1 macrophages that infiltrate to adipose tissue and lead to dysfunction and inflammation [9]. In macrophages, L5 LDL induces granulocyte colony-stimulating factor (G-CSF) and GM-CSF overproduction [84,85]. These biomarkers are associated with inflammation, increased risk of cardiovascular complications and STEMI [85,86]. Besides, L5 LDL enhances the overexpression of interleukin (IL)-1β (IL-1β) through the activation of the nucleotide-binding oligomerization domain (NOD)-like receptor pyrin domain containing 3 (NLRP3) inflammasomes [87]. 

## 4. Clinical Significance of L5 LDL

### 4.1. Cardiometabolic Disorders

In healthy subjects, the LDL constitution comprises mostly L1 LDL and the least of L5 LDL. The majority of their LDL, L1 LDL, is endocytosed by the low-density lipoprotein receptor (LDL-R) and processed by endolysosomes to provide nutrients to the cells [57]. In contrast, L5 LDL is the atherogenic component and its levels are elevated in the plasma of patients with increased cardiovascular risk [7,9,10]. The elevated levels of plasma L5 LDL can be found in patients with CAD, hyperlipidemia (HLP) [7], metabolic syndrome (MetS) [9], familial hypercholesterolemia (FH) [88], diabetes mellitus (DM) [8], and in smokers [89]. The reference range of L5% in total LDL and absolute L5 concentration ([L5] = L5% × LDL-C) for healthy adults were determined to be less than 1.6% and less than 1.7 mg/dL [7]. The increasing levels of L5% and absolute L5 concentration (mg/dL) can be found in patients mentioned above, respectively (Table 1). The level of absolute L5 concentration of STEMI patients could be 11.1 ± 14.0 times higher than in the healthy controls [10]. According to previous studies, L5 LDL can disrupt the integrity of ECs through LOX-1 [8] and promote the cell apoptosis by activating the signaling cascade downstream of CD14/TLR4 [80]. Hence, lowering the levels of L5 LDL could further be the goal of treatment in those diseases.

### 4.2. Acute Ischemic Events

According to the American Stroke Association (ASA) statement, acute ischemic stroke stays as the fifth cause of death and a leading cause of disability in the United States. Previous studies showed that L5% and L5 concentration in healthy control are 0.5 ± 0.3 and 0.5 ± 0.4 mg/dL, respectively. Nevertheless, in stroke patients, these markers will be elevated as 19.1 ± 10.6 and 20.6 ± 13.5 mg/dL, respectively [11]. L5 LDL can further reduce the viability of cultured ECs [8,20,22] and promote EC dysfunction by triggering procoagulant activity. Even more, L5 LDL may increase EC-platelet interactions to induce platelet activation [10,75]. On the basic concept of ASAs, 80 percent of stroke events are preventable. Early diagnosis may be relevant to the prevention of stroke. Overall, L5 LDL plays a critical role in the development of stroke. The ablation of L5 LDL may be a compelling goal of treatment in stroke.

### 4.3. Autoimmune Diseases

Patients with systemic lupus erythematosus (SLE) frequently accompanied by early vascular aging (EVA) [12] and severe atherosclerosis complications [90,91,92], though their low-density lipoprotein (LDL) levels remain low. Comparing LDL-C and L5 LDL levels between controls and patients, results showed that LDL-C was lower in SLE patients than in controls (105.1 ± 32.3 mg/dL versus 118.2 ± 23.3 mg/dL), but the concentration of L5 LDL in SLE patients were three times higher than in controls (2.4 ± 1.3 mg/dL versus 0.8 ± 0.4 mg/dL) [12]. 

As per SLE, the plasma L5% and L5 levels were significantly higher in rheumatoid arthritis (RA) patients than in controls. The expression levels of LDLR in PBMCs were no significant difference in observed, but the levels of LOX-1 in PBMCs of RA patients were two to three times higher than healthy controls [13]. This phenomenon indicates that the higher expression levels of LOX-1 and L5 LDL, the more can be uptake through LOX-1 to cause ECs dysfunction and atherosclerosis.

## 5. Implication of L5 LDL

### 5.1. Diagnostic Value of L5 LDL

According to the 2020 report from the American Heart Association (AHA), cardiovascular diseases remain the leading cause of death worldwide [93]. Although several new drugs and more aggressive approaches are proposed [2,94], the laboratory identification of atherogenic factors is still not specific. Currently, the clinical evaluation of ASCVD risk is based on (1) HDL-C in men: [<40 mg/dL]; in women: [<50 mg/dL]; (2) LDL-C [>160 mg/dL]; (3) TG [≥200 mg/dL]; (4) cholesterol ratio, i.e., total cholesterol/HDL. Other important issues include who should be treated, dosage, and goal of treatment. 

Due to the lack of a quick and feasible measurement, now estimating the 10-year ASCVD risk by the Estimator App (developed by the American College of Cardiology Foundation) is essential. Several factors are calculated, including age, gender, blood pressure, total cholesterol, HDL, LDL, histories such as diabetes, smoking, statin, and aspirin usage. Recent ACC/AHA 2018 cholesterol guidelines for the prevention of cardiovascular diseases adopted the risk-enhancers concept, focusing on more detailed patients’ history, including metabolic syndrome, chronic kidney disease, inflammatory condition, premature menopause, high-risk race, and some other new lipid parameters and biomarkers such as high sensitivity C-reactive protein (hs-CRP), Lipoprotein (a) (Lp(a)), apoB100, and ankle-brachial index (ABI) [95] in sharing decision-making.

Our recent clinical studies show that the four statin benefit groups are characterized by higher levels of L5 LDL [14]. L5 LDL markers, either percentage or the absolute plasma concentration, can be more reliable than those listed above (Table 1) [7,8,9,10,11,12,13,15,67]. Besides, the molecular mechanisms and the clinical relevance of L5 LDL provide strong evidence-based supports to demonstrate that L5 LDL can be potential biomarkers in the early diagnosis of vascular aging, atherosclerosis and cardiovascular diseases [6,24,25,28,55,56,60,63,70,84]. 

Based on the reference range of L5 LDL and the fact that higher concentration increases the risks of ASCVD, we propose that individuals with absolute plasma L5 LDL level of less than 1.7 mg/dL are within the acceptable safe range. These individuals should avoid lipid-lowering therapy despite that their LDL-C levels might exceed 190 mg/dL or above. For patients with increasing plasma L5 LDL levels, the screening tests for CVD such as carotid intima-media thickness, pulse wave velocity, and ankle-brachial index for peripheral artery disease, exercise stress test, and multidetector computerized tomography (MDCT) for CAD are highly recommended. 

For those patients with established clinical ASCVD, acute coronary syndrome, CAD with percutaneous coronary intervention or bypass surgery, the therapeutic goal of absolute L5 LDL should still less than 1.7 mg/dL, even if their LDL-C has been lowered to less than 70 mg/dL that recommended by current guideline. In fact, the LDL-C goal has been revised to less than 55 mg/dL for patients with very-high risk and to 40 mg/dL for patients with recurrent myocardial infarction within two years by recent ESC/EAS 2019 dyslipidemia guideline. It reflects that these patients are of high toxic lipid components and these patients carry poor cardiovascular outcomes. 

By keeping L5 LDL goal <1.7 mg/dL, more aggressive lipid-lowering agents, e.g., ezetimibe or PSCK9 inhibitors, on top of maximally tolerated statin use, should be considered regardless of the LDL-C level. That is why the 2013 ACC/AHA cholesterol guideline emphasized the intensity instead of the LDL-C target for four statin-benefit groups. Notably, current lipid-lowering agents available have not been shown to specifically reduce the toxic L5 LDL, but lower the overall LDL-C. Targeting specifically on toxic L5 LDL therapeutic strategies has been developing and hopefully can be beneficial for better CV outcomes in the near future. 

### 5.2. Clinical Implication of L5 LDL

“No treatment goal”, “the lower the LDL-C, the better”, and “beyond absolute goals toward personalized risk” are the current strategies of primary and secondary prevention of CVDs [96,97]. The incidence of subclinical atherosclerosis remains as high as 64% in the LDL-C range of 150~160 mg/dL and 11% in the range of 60~70 mg/dL [4,98]. However, patients receiving statin may complain about the side effects of muscle pain or weakness; and some of them stopped taking statins because of the intolerance of side effects [99,100]. 

**Figure 2:**
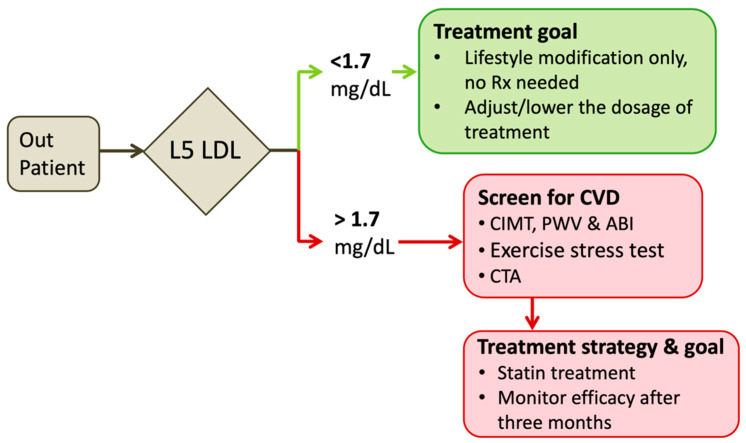
Reference ranges of L5 LDL and the strategy of statin treatment. Examining the plasma levels of L5 LDL can be useful to determine (1) whether to receive statin or not, (2) more detailed physical exams for cardiovascular functions and lesions, (3) the treatment strategy and goal. Rx: medical prescription; CIMT: carotid intima-media thickness; PWV: pulse wave velocity; ankle-brachial index; CTA: computed tomography angiography.

Based on ROC curve analysis, a L5 LDL level of more than 1.7 mg/dL is highly associated with subclinical atherosclerosis or increased odds ratio of CVDs [7,13]. We propose that individuals with a plasma L5 LDL level of more than 1.7 mg/dL are required for intervention. For example, statin therapies may be beneficial in reducing the electronegative subfraction of LDL [14,15,101,102]; while three months after discontinuation, the concentration of L5 LDL may return to the untreated-baseline levels [15]. Besides, the treating goal of LDL-lowering therapies should be the L5 LDL concentration of less than 1.7 mg/dL (Figure 2).

### 5.3. Drawback and Limitation of L5 LDL Quantification

Even though the clinical significance of L5 LDL can be high, some drawbacks and limitations must be mentioned. First of all, a large scale of clinical study must be done to validate the significance of being a novel clinical biomarker. Dr. Sanchez-Quesada and many other study groups published a variety of papers mentioning the clinical prevalence and the pathogenic mechanisms of electronegative LDL [103,104,105,106,107,108]. The level difference between patients and controls are comparable, and the atherogenic properties are noticeable. However, since there are algorithm differences in both experimental materials and isolation protocols, it would be necessary to adjust the reference range of controls. Moreover, due to the laborious work and long turn-around time (Figure 1), the diagnostic method’s efficiency must be significantly improved. A rapid and clinically feasible diagnostic approach must be invented. Moreover, a precision medicine targeting the removal of L5 LDL is of great clinical importance.

## 6. Conclusions

In summary, the recognition of L5 LDL keeps rising. Until now, it has been a promising clinical biomarker for cardiovascular diseases despite the levels of LDL-C. We propose that a large-scale population survey and a high throughput methodology are required. Undisputedly, new strategies for directly eliminating L5 LDL from the bloodstream are essential works in the future.

## Figures and Tables

**Table 1:** Clinical significance of electronegative low-density lipoprotein (L5 LDL)–data collected in Taiwan.

Publications	*Sci Rep* [7]	*JCEM* [9]	*Blood* [10]	*Blood* [11]	*JCM* [13]	*AR* [12]
**Subjects**	HLP	MetS	STEMI	stroke	RA	SLE
n	35	29	30	35	30	45
T-CHOL	235.9 ± 36.6	232.9 ± 31.6 ^a^	179.1 ± 33.9	151.4 ± 34.3	219 (193–245) ^c^	NA
TG	164.5 ± 90.6	259.6 ± 209.1 ^a^	119.6 ± 65.6	123.8 ± 72.5	123 (87–170) ^c^	NA
HDL-C	53.1 ± 16.4	45.4 ± 9.7 ^a,^^**^	38.5 ± 8.6 ^***^	32.7 ± 6.6	58.5 (48–66) ^c^	48.9 ± 17.5 ^**^
LDL-C	146.0 ± 34.9 ^***^	142.2 ± 41.8 ^a^	116.7 ± 32.4	105.4 ± 34.5	142 (111–168) ^c^	105.1 ± 32.3 ^*^
L5%	2.3 ± 1.3 ^***^	5.3 ± 6.9 ^a,***^	15.4 ± 14.5 ^***^	19.1 ± 10.6 ^***^	2.0 (1.3–4.5) ^c,^^***^	2.4 ± 1.3 ^***^
[L5]	3.2 ± 2.0 ^***^	7.3 ± 9.8 ^a^^,^^**^	18.9 ± 21.0 ^***^	20.6 ± 13.5 ^***^	2.9 (1.7–5.7) ^c,^^***^	2.4 ± 1.3 ^***^
**Controls**	NHC	None-MetS	NHC	NHC	NHC	NHC
n	35	29	30	25	12	37
T-CHOL	173.4 ± 32.8	215.3 ± 50.8 ^b^	179.3 ± 32.9	150.8 ± 32.9	208 (201–231) ^c^	NA
TG	79.7 ± 56.1	91.6 ± 47.5 ^b^	78.6 ± 59.8	109 ± 38.5	90 (72.8–126) ^c^	NA
HDL-C	54.4 ± 14.0	56.5 ± 17.4 ^b^	55.6 ± 14.2	41.8 ± 12.1	59 (46–78) ^c^	58 ± 16
LDL-C	103.3 ± 27.6	140.9 ± 44.5 ^b^	108.1 ± 28.4	92.6 ± 33.5	131 (120–155) ^c^	118.2 ± 23.3
L5%	1.3 ± 0.7	2.1 ± 1.4 ^b^	1.5 ± 1.1	0.5 ± 0.3	0.9 (0.6–1.1) ^c^	0.7 ± 0.3
[L5]	1.3 ± 0.7	3.0 ± 2.0 ^b^	1.7 ± 1.5	0.5 ± 0.4	1.3 (0.8–1.5) ^c^	0.8 ± 0.4
L5% [P’t-NHC]	1.0 ± 0.2	3.2 ± 1.3	13.9 ± 2.7	18.6 ± 1.8	NA	1.7 ± 0.2
[L5] [P’t-NHC]	1.9 ± 0.4	4.3 ± 1.9	17.2 ± 3.8	20.1 ± 2.3	NA	1.6 ± 0.2

Data are presented as the mean ± SD unless indicated otherwise. * *p* < 0.05; ** *p* < 0.01; *** *p* < 0.001; ^a^ Patient who met criteria of metabolic syndrome (MetS); ^b^ Individual who met two or fewer criteria; ^c^ Data are presented as the median (interquartile range). HLP: hyperlipidemia; STEMI: ST-segment elevation myocardial infarction; RA: rheumatoid arthritis with subclinical atherosclerosis; SLE: systemic lupus erythematosus; NHC: normal healthy control; T-CHOL: total cholesterol; TG: triglyceride; HDL-C: high-density lipoprotein cholesterol; LDL-C: low-density lipoprotein cholesterol; NA: not available; L5%: percentage of L5 subfraction in total LDL; [L5]: L5 concentration; L5% [P’t – NHC]: the difference of L5% between two groups; [L5] [P’t – NHC]: the difference of [L5] between two groups.
